# Association between Caffeine Consumption in Pregnancy and Low Birth Weight and Preterm Birth in the birth Cohort of Ribeirão Preto

**DOI:** 10.1055/s-0038-1675806

**Published:** 2018-12

**Authors:** Fernanda Pino Vitti, Carlos Grandi, Ricardo de Carvalho Cavalli, Vanda Maria Ferreira Simões, Rosângela Fernandes Lucena Batista, Viviane Cunha Cardoso

**Affiliations:** 1 Department of Pediatrics, Faculdade de Medicina de Ribeirão Preto da Universidade de São Paulo, Ribeirão Preto, SP, Brazil; 2 Department of Pediatrics, Facultad de Medicina de la Universidad de Buenos Aires, Argentina; 3 Department of Gynecology and Obstetrics, Faculdade de Medicina de Ribeirão Preto da Universidade de São Paulo, Ribeirão Preto, SP, Brazil; 4 Department of Public Health, Universidade Federal do Maranhão, São Luiz, MA, Brazil

**Keywords:** caffeine, pregnancy, cohort studies, low birth weight, preterm birth, cafeína, gestação, estudo de coorte, baixo peso ao nascer, nascimento pré-termo

## Abstract

**Objective** To describe caffeine consumption during pregnancy and its association with low birth weight (LBW) and preterm birth in the birth cohort of Ribeirão Preto, state of São Paulo, Brazil, in 2010.

**Methods** Cohort study, with descriptive and analytical approach. Data included 7,607 women and their newborns in Ribeirão Preto, state of São Paulo, Brazil. The women answered standardized questionnaires about reproductive health, prenatal care, life habits, sociodemographic conditions, and information about coffee intake. The independent variable was high caffeine consumption (≥300 mg/day) from coffee during pregnancy, and the dependent variables were LBW (birth weight < 2,500 g) and preterm birth (< 37 weeks of gestational age). Four adjusted polytomous logistic regression models, relative risk (RR) and 95% confidence interval (CI) were fitted: biological and sociodemographic conditions; obstetric history; current gestational conditions; and all variables included in the previous models.

**Results** A total of 4,908 (64.5%) mothers consumed caffeine, 143 (2.9%) of whom reported high consumption. High caffeine intake was significantly associated with reduced education and with the occupation of the head of the family, nonwhite skin color, not having a partner, higher parity, previous abortion and preterm birth, urinary tract infection, threatened abortion, alcohol consumption and smoking. No association was found between high caffeine consumption and LBW or preterm birth in both unadjusted (RR = 1.45; 95% CI: 0.91–2.32; and RR = 1.16; 95% CI: 0.77–1.75, respectively) and adjusted analyses (RR = 1.42; 95% CI: 0.85–2.38; and RR = 1.03; 95% CI: 0.65–1.63, respectively).

**Conclusion** In this cohort, high caffeine intake was lower than in other studies and no association with LBW or preterm birth was found.

## Abstract (pt)

**Resumo:**

**Objetivo** Descrever a associação entre consumo de cafeína durante a gestação com baixo peso ao nascer (BPN) e nascimento pré-termo (PT) na coorte de Ribeirão Preto, estado de São Paulo, Brasil, em 2010.

**Métodos** Estudo de coorte, com abordagem descritiva e analítica. Foram incluídas 7.607 mulheres e seus recém-nascidos em Ribeirão Preto, São Paulo, Brasil. As mulheres responderam a questionários padronizados sobre saúde reprodutiva, cuidados pré-natais, hábitos de vida, condições sociodemográficas e consumo de cafeína. A variável independente foi alto consumo de cafeína (≥300 mg/dia) durante a gestação e as dependentes foram BPN (peso < 2.500 g) e nascimento PT (< 37 semanas de gestação). Foram calculados riscos relativos (RRs) e intervalos de confiança (ICs) de 95% em quatro modelos de regressão logística: condições biológicas e sociodemográficas; história obstétrica; condições da gestação atual; e todas as variáveis incluídas nos modelos anteriores.

**Resultados** Um total de 4.908 (64,5%) mães consumiram cafeína, e destas, 143 (2,9%) relataram alto consumo. Alto consumo de cafeína esteve associado com menor escolaridade materna, ocupação do chefe da família, cor de pele não branca, mulheres sem companheiro, maior paridade, aborto e nascimento PT anterior, infecção do trato urinário, ameaça de aborto, consumo de álcool e tabagismo. Não foi encontrada associação entre alto consumo de cafeína e BPN ou nascimento PT nas análises não ajustada (RR = 1,45; IC 95%: 0,91–2,32; e RR = 1,16; IC 95%: 0,77–1,75, respectivamente) e ajustada (RR = 1,42; IC 95%: 0,85–2,38; e RR = 1,03; IC 95%: 0,65–1,63, respectivamente).

**Conclusão** Nessa coorte, o alto consumo de cafeína foi menor que em outros estudos e não foi encontrada associação com BPN ou nascimento PT.

## Introduction

Maternal exposures, such as excessive caffeine consumption during pregnancy, are likely associated to low birth weight (LBW) and preterm birth. This issue has been the subject of several epidemiological studies.^1^ ^2^ ^3^ ^4^ However, the results of these studies are contradictory, which is partly explained by the heterogeneity in study design, by the measurement of caffeine intake, and by the identification of confounders.^3^ ^5^ ^6^ ^7^

Caffeine (1,3,7- trimethylxanthine), an alkaloid contained in coffee, tea, cocoa, soft drinks with cola, some medications, and stimulants, is a substance generally socially accepted in the whole world that acts as a psychoactive stimulant.^1^ In pregnant women, caffeine passes the placenta freely, where the immature fetal liver enzyme CYP3A4 is unable to metabolize it,^8^ resulting in the accumulation of caffeine in the fetus;^9^ ^10^ ^11^ substantial quantities of caffeine pass into the amniotic fluid and into the umbilical cord blood, being distributed to all of the tissues of the fetus. Therefore, this may increase the concentration of cellular cyclic adenosine monophosphate (cAMP), interfering in fetal cell growth and development.^12^ Moreover, caffeine causes most of its biological effects via antagonizing all types of adenosine receptors and increasing epinephrine concentrations in the mother and in the fetus, resulting in decreased placental blood flow and hypoxia.^13^

Studies showed that the amount of caffeine intake during pregnancy is relatively high. The prevalence in women who had preterm birth was 68.1%,^5^ whereas, in Brazil, the prevalence was 75% during pregnancy.^14^

Several studies showed an increased risk of adverse perinatal outcomes when caffeine consumption was ≥300 mg/day,^1^ ^9^ ^15^ between 150 and 300 mg/day,^16^ or < 150 mg/day.^5^ Other studies did not find any association,^17^ ^18^ but most of the studies consider high caffeine intake as ≥300 mg/day.^1^ ^9^ ^15^ ^19^ ^20^ ^21^ ^22^

In Ribeirão Preto, state of São Paulo, Brazil, a birth cohort started in January 2010, which included 7,702 mothers and newborns (NB) in facilities right after childbirth. Cohort studies are considered paramount in understanding the effects of intrauterine life and of childhood conditions on adult health.^23^

Thus, the objectives of the present study were to describe caffeine intake during pregnancy and estimate the risks between high caffeine consumption with LBW and preterm birth, on the 2010 birth cohort of Ribeirão Preto, state of São Paulo, Brazil.

## Methods

This cohort is part of the Brazilian Ribeirão Preto and São Luís Birth Cohort Studies (BRISA, in the Portuguese acronym,), with the main objectives of evaluating new preterm birth risk factors (neuroendocrine, immunoinflammatory, and medical intervention hypothesis), perinatal health indicators, and impact on later preterm birth growth. The cohort included 7,702 mothers and their NBs between January 1^st^ and December 31^th^, 2010; 95 twin pregnancies were excluded, remaining 7,607 mothers and NBs evaluated.

Childbirth took place in eight maternity hospitals of Ribeirão Preto and, during the study period, trained personnel visited these facilities to interview the mothers and collect information about the NBs from medical records.

The dependent variables were low birth weight (LBW) (birth weight [BW] < 2,500 g) and preterm birth (gestational age < 37^+0^ weeks), and the independent variable was caffeine intake (only from coffee). Information about daily and weekly frequency of caffeine consumption during each trimester of gestation (days/week), and type of container used were collected. No information about tea, chocolates, sodas and other sources of caffeine were recorded. Caffeine consumption from coffee during pregnancy was categorized as none, low (< 300 mg/day) and high (≥300 mg/day), according to the Care Study Group.^24^ The daily frequency of caffeine consumption in milliliters was calculated by multiplying the daily quantity (times/day) by the daily frequency (days/week) and the type of container (50 or 200 ml) in each trimester. Then, the result was divided by 7 days of the week. Since 125 ml of coffee corresponded to 90 mg of caffeine,^7^ each ml of coffee intake contained 0.72 mg of caffeine.

The questionnaires included mother variables (demographic, social and reproductive health, life habits, pregnancy complications, and coffee intake) and NB variables (gender, BW, gestational age [GA], morbidities, and stillborn). Newborn anthropometry included BW and length.

Maternal age was categorized as ≤ 19, between 20 and 34, and ≥ 35 years old. Maternal education was categorized as ≤ 8, between 9 and 11, and ≥ 12 years of study. Skin color was self-referenced as either white or not white. Marital status was categorized as with or without partner. The occupation of the head of the family^25^ was categorized as no manual, qualified, semi qualified, and not qualified workers. Parity was categorized as 1, between 2 and 3, and ≥ 4 childbirths. Information was collected regarding previous preterm birth, abortion, and stillbirth; hypertension, diabetes, urinary tract infection and odontological treatments during pregnancy; threatened abortion and preterm delivery; as well as alcohol consumption and maternal smoking.

For improving the presentation of the results, we followed the strengthening the reporting of observational studies in epidemiology **(**STROBE) initiative.^26^

Data analysis included mean (standard deviation [SD]), median (interquartile range [IQR]) or proportions (95% confidence interval [CI]). A univariate risk analysis between LBW and preterm birth, high caffeine consumption and covariates were performed. Then, four adjusted polytomous logistic regression models (relative risk [RR] and 95% confidence interval [95% CI]) were fit. The first one was adjusted for biological and sociodemographic conditions (age, maternal education, skin color, marital status, occupation of the head of the family); the second included obstetric history (parity, previous preterm birth, abortion and stillbirth); the third was adjusted for current gestational conditions (gestational hypertension and diabetes, threatened abortion and preterm delivery, alcohol consumption, maternal smoking, urinary tract infection, and odontological treatments during pregnancy); and the last was adjusted for all variables included in the previous models. The reference value was the absence of caffeine consumption. The goodness of fit was tested using the Hosmer-Lemeshow test. All analyses were performed with Stata software, version 13.0 (Stata Corp., College Station, TX, USA) and SAS software, version 9.2 (SAS Institute, Cary, NC, USA). Statistically significant difference was set at *p* < 0.05. The present study was approved by the Research Ethic Committee of the Ribeirão Preto Medical School of the Universidade de São Paulo (process n° 10400/2012), and all participants received and signed the informed consent form.

## Results

A total of 7,607 mothers and their NBs were included. Of these, 4,908 (64.5%; 95% CI: 63–65) consumed caffeine from coffee during pregnancy, 143 of whom (2.9%; 95% CI: 25–34) ingested ≥300 mg/day. The mean BW was 3,143 g (SD = 553); 8.7% were LBW, and 1.7% were very low birth weight (VLBW [< 1,500 g]). The mean GA was 285 (SD = 104) days, and 14.5% of the childbirths were preterm (< 259 days).

The majority of the total population studied ingested up to 500 mg of caffeine per day, with a median caffeine intake of 91.5 mg/day (IQR = 143.8); there was no difference per trimester of caffeine intake (data not shown).

High caffeine intake was significantly associated with reduced maternal education, occupation of the head of the family, nonwhite skin color, and not having a partner (Table 1).

**Table 1:** Selected sociodemographic maternal characteristics of the Ribeirão Preto Birth Cohort of 2010 in terms of caffeine consumption

Characteristic	< 300 mg/day *n* (%)	≥300 mg/day *n* (%)	*p-value*
Maternal age (years old)			0.153
20–34	3,543 (74.4)	111 (77.6)	
< 20	526 (11.0)	19 (13.3)	
≥35	696 (14.6)	13 (9.1)	
Maternal education (years)*			< 0.001
≥ 12	1,099 (23.6)	8 (5.7)	
≤ 8	1,197 (25.7)	76 (54.7)	
9–11	2,358 (50.7)	55 (39.6)	
Occupation of the head of the family*			< 0.001
No manual workers	711 (15.4)	2 (1.5)	
Qualified workers	702 (15.2)	8 (5.9)	
Semi-qualified workers	2,072 (45.0)	68 (50.4)	
Not qualified workers	1,122 (24.4)	57 (42.2)	
Skin Color*			< 0.001
White	2,814 (59.5)	56 (40.3)	
Not white	1,915 (40.5)	83 (59.7)	
Marital status*			< 0.001
With partner	4,182 (87.8)	105 (73.4)	
Without partner	581 (12.2)	38 (26.6)	
Total	4,765 (100)	143 (100)	

*Excluded cases.

High caffeine intake was significantly associated with higher parity, previous abortion and preterm birth, urinary tract infection, threatened abortion, alcohol consumption, and smoking (Table 2).

**Table 2:** Selected obstetric characteristics of the Ribeirão Preto Birth Cohort of 2010 in terms of caffeine consumption

Characteristic	< 300 mg/day *n* (%)	≥300 mg/day *n* (%)	*p*-value
Parity (childbirths)*			< 0.001
1	2,242 (47.1)	41 (28.7)	
2–3	2,152 (45.2)	69 (48.2)	
≥4	364 (7.7)	33 (23.1)	
Previous abortion*			0.023
No	3,859 (81.0)	105 (73.4)	
Yes	903 (19.0)	38 (26.6)	
Previous preterm birth*			0.003
No	4,079 (86.5)	109 (78.4)	
Yes	639 (13.5)	30 (21.6)	
Previous stillbirth*			0.404
No	4,688 (98.4)	142 (99.3)	
Yes	75 (1.6)	1 (0.7)	
Gestational hypertension*			0.680
No	4,163 (87.7)	127 (88.8)	
Yes	586 (12.3)	16 (11.2)	
Gestational diabetes*			0.999
No	4,450 (93.7)	134 (93.7)	
Yes	299 (6.3)	9 (6.3)	
Urinary tract infection*			0.049
No	3,256 (68.6)	87 (60.8)	
Yes	1,489 (31.4)	56 (39.2)	
Odontological treatments*			0.222
No	3,315 (70.7)	93 (66.0)	
Yes	1,373 (29.3)	48 (34.0)	
Threatened abortion*			0.049
No	4,427 (93.0)	127 (88.8)	
Yes	329 (7.0)	16 (11.2)	
Threatened preterm birth*			0.275
No	4,129 (86.9)	119 (83.8)	
Yes	620 (13.1)	23 (16.2)	
Alcohol consumption*			< 0.001
No	3,578 (75.1)	86 (60.1)	
Yes	1,185 (24.9)	57 (39.9)	
Smoking			< 0.001
No	4,213 (88.4)	88 (61.5)	
Yes	552 (11.6)	55 (38.5)	
TOTAL	4,765 (100)	143 (100)	

*Excluded cases.

Low birth weight was the only condition significantly associated with high caffeine consumption (Table 3).

**Table 3:** Selected neonatal characteristics of the Ribeirao Preto Birth Cohort of 2010in terms of caffeine consumption

Characteristic	< 300 mg/day *n* (%)	≥300 mg/day *n* (%)	*p*-value
Gender*			0.928
Male	2,394 (50.3)	72 (50.3)	
Female	2,366 (49.7)	71 (49.6)	
Low birth weight			0.034
No	4,370 (91.7)	124 (86.7)	
Yes	395 (8.3)	19 (13.3)	
Preterm Birth			0.424
No	4,079 (85.6)	119 (83.2)	
Yes	686 (14.4)	24 (16.8)	
Congenital Malformations*			0.290
No	4,701 (98.9)	140 (97.9)	
Yes	54 (1.1)	3 (2.1)	
Total	4,765 (100)	143 (100)	

Low birth weight: birth weight < 2500 g. Preterm birth: gestational age < 259 days. *Excluded cases.

Crude risks for LBW and preterm birth were 1.45 (95% CI: 0.91–2.32) and 1.16 (95% CI: 0.77–1.75), respectively. For LBW, the RR increased slightly after the adjustment, but no differences were observed (Table 4). For preterm birth, the RR decreased slightly after the adjustment in the four models, but without association with high caffeine intake during pregnancy (Table 5).

**Table 4:** Crude and adjusted risks of low birth weight, according to caffeine consumption. Ribeirão Preto Birth Cohort of 2010

Caffeine consumption	Crude Relative Risk	Adjusted Relative Risk			
		Model 1	Model 2	Model 3	Model 4
	RR (95% CI)	RR1 (95% CI)	RR2 (95% CI)	RR3 (95% CI)	RR4 (95% CI)
Not consumed	1	1	1	1	1
< 300 mg/day	0.91 (0.77–1.06)	1.10 (0.93–1.32)	1.07 (0.91–1.27)	1.08 (0.91–1.27)	1.10 (0.92–1.32)
≥ 300 mg/day	1.45 (0.91–2.32)	1.52 (0.92–2.52)	1.56 (0.97–2.50)	1.34 (0.53–2.16)	1.42 (0.85–2.38)

Abbreviations: CI, confidence interval; RR, relative risk.

Model 1 adjusted for maternal age, education and skin color, marital status, and occupation of the head of the family.

Model 2 adjusted for parity, previous preterm birth, abortion, and stillbirth.

Model 3 adjusted for gestational hypertension and diabetes, threatened abortion and preterm delivery, alcohol consumption, maternal smoking, and urinary tract infection.

Model 4 adjusted for all variables included in the previous models.

**Table 5:** Crude and adjusted risks of preterm birth according to caffeine consumption. Ribeirão Preto Birth Cohort of 2010

Caffeine consumption		Crude Relative Risk	Adjusted Relative Risk			
			Model 1	Model 2	Model 3	Model 4
		RR (95% CI)	RR1 (95% CI)	RR2 (95% CI)	RR3 (95% CI)	RR4 (95% CI)
Not consumed		1	1	1	1	1
< 300 mg/day		0.99 (0.87–1.13)	1.07 (0.94–1.22)	1.10 (0.97–1.25)	1.11 (0.97–1.26)	1.10 (0.96–1.26)
≥ 300 mg/day		1.16 (0.77–1.75)	1.16 (0.74–1.80)	1.12 (0.73–1.69)	1.12 (0.72–1.73)	1.03 (0.65–1.63)

Abbreviations: CI, confidence interval; RR, relative risk.

Model 1 adjusted for maternal age, education and skin color, marital status, and occupation of the head of the family.

Model 2 adjusted for parity, previous preterm birth, abortion, and stillbirth.

Model 3 adjusted for gestational hypertension and diabetes, threatened abortion and preterm delivery, alcohol consumption, maternal smoking, urinary tract infection and odontological treatments during pregnancy.

Model 4 adjusted for all variables included in the previous models.

## Discussion

In the present study, 64.5% of the pregnant women drank coffee and there was no difference per trimester of caffeine intake. Only 2.9% of these women reported high caffeine intake, without association with LBW or preterm birth.

Similarly to the present study, large studies that incorporated trimester-specific questionnaires showed no difference between the trimesters.^24^

In other studies, the incidence of high caffeine consumption was higher than in Ribeirão Preto (18.9%,^11^ 13.5%,^9^ 12%,^2^ and 8.3%^27^), possibly by including other sources of caffeine. Another explanation is that when other health risks are present, doctors might discourage caffeine consumption, resulting in a lower coffee intake in contrast with the healthier women. This is supported by the elevated rate of gestational hypertension, diabetes, and urinary tract infection observed in the studied population.

Caffeine consumption from coffee, tea, soft drinks with cola, chocolate, and some medications is frequently described,^19^ including in pregnant women. Previous studies found that a high caffeine intake could be harmful during pregnancy because caffeine crosses the placenta and accumulates in the fetus, decreases blood flow and produces biochemical changes, with smaller BW or shorter GA, and increases abortions and the risk of congenital anomalies.^10^ ^13^

In the present cohort, high caffeine consumption during pregnancy was not associated with LBW or preterm birth, in crude and adjusted models. To study the associations between the independent variables and LBW and preterm birth, odds ratios (ORs) were estimated by using a hierarchized (polytomous) logistic regression model.

Other studies reinforced this finding;^2^ ^3^ ^6^ ^11^ ^17^ ^28^ ^29^ ^30^ recently, two meta-analyses reported a higher risk of delivering LBW newborns, while no association with preterm delivery was found.^31^ ^32^

However, some authors found an association with different caffeine levels (consumption between 70–92 mg/day,^30^ > 300 mg/day,^16^ ^21^ ^22^ ^33^ ≥140 mg/day,^9^ and ≥ 6 cups, with 90 mg/cup),^7^ but most of the associations were found with the highest levels of consumption.^31^

Associations between lower values of caffeine intake and LBW were found in some studies, which divided the cutoff points into between 1 and 100 mg/day, between 101 and 200 mg/day, and between 201 and 300 mg/day.^16^ ^21^

The only existing randomized controlled trial that studied the effect of caffeine on a Danish cohort of 1,207 women during the second half of pregnancy observed no effect on BW or GA at birth.^34^ Another case control study with 502 mothers did not find any association between caffeine intake and preterm birth.^3^

Several recognized maternal conditions were statistically associated with LBW and preterm birth, such as not having a partner, highest parity, previous preterm birth or stillbirth, gestational hypertension, threatened preterm birth, and smoking, in accordance with other studies.^27^ ^28^

High caffeine intake was significantly associated with reduced education, occupation of the head of the family, nonwhite skin color, not having a partner, higher parity, previous abortion and preterm birth, urinary tract infection, threatened abortion, alcohol consumption, and smoking.

Pacheco et al^27^ found that high caffeine intake was associated with reduced education, higher parity, alcohol consumption, and smoking. No association with age, previous preterm birth, and body mass index (BMI) was found.

Another study with 7,025 women found similar results: women without partner, reduced education, higher parity, history of preterm birth, and smoking presented a higher caffeine intake.^30^

The main differences with other studies were the heterogeneity of the caffeine exposure in different concentrations, of the diversity of brands, of the preparation according to each region and country, of the different methods for the assessment of caffeine consumption (postpartum questionnaires or caffeine concentration in plasma and in saliva measured by swab), of the inadequate measures of caffeine intake, of the insufficient statistical power and control of confounding variables. In some studies, higher consumption of caffeine intake was found with a similar cutoff (> 300 mg/day), but including tea, chocolate and other sources of caffeine.^15^ ^27^

Some studies suggest a possible effect of chemicals present in coffee and tea other than caffeine, such as polyphenols, which contribute to adverse pregnancy outcomes including preterm delivery and preeclampsia.^35^ Therefore, studies investigating the effect of caffeine tend to overlook the fact that other components in coffee and tea may be contributing factors.

Several limitations of the present study should be mentioned. First, the sample size was originally not computed for caffeine consumption and, therefore, a type I error cannot be ruled out. Second, caffeine intake during pregnancy was measured on a self-reported basis only after birth, a fact known to underestimate the frequency and amount of caffeine ingested by pregnant women; consequently, our study is subjected to recall bias. The information about caffeine intake were obtained by questionnaires; therefore, it was not possible to quantify neither the concentration, the brand of coffee, nor the method of preparation (strong, medium, weak). The questionnaires employed did not have information about other sources of caffeine, such as tea, soft drinks with cola, chocolate, or some medications, which can influence the lower prevalence of consumption of caffeine compared with the aforementioned studies. Third, the present study was performed in a cohort of relatively healthy pregnant women with singleton pregnancies. Therefore, the findings can only be generalized to an obstetric population with a similar risk of complications. Finally, we cannot exclude the possibility of residual confounding variables.

The strength of the present study was the assessment of a birth cohort followed-up since the prenatal period with a high response rate (96%). Since the data were collected at two different periods, we were able to confirm the information and to adjust the outcomes for a wide range of known confounders, such as maternal behaviors and sociodemographic factors, and possible risk factors of adverse perinatal outcomes could be registered.

Therefore, more detailed studies analyzing sources of caffeine are necessary, and also to verify if any specific trimester of gestation can be more vulnerable to caffeine exposure.

## Conclusion

High caffeine intake during pregnancy was not associated with an increased risk of LBW and preterm risk in the birth cohort of Ribeirão Preto.
